# Impact of aquaporin-4 and CD11c + microglia in the development of ependymal cells in the aqueduct: inferences to hydrocephalus

**DOI:** 10.1186/s12987-024-00548-2

**Published:** 2024-07-02

**Authors:** Francisco Mayo, Lourdes González-Vinceiro, Laura Hiraldo-González, Francisco D. Rodríguez-Gómez, Claudia Calle-Castillejo, Manuel Mayo, Vanina Netti, Reposo Ramírez-Lorca, Miriam Echevarría

**Affiliations:** 1 Instituto de Biomedicina de Sevilla, IBiS/Hospital Universitario Virgen del Rocío/CSIC/Universidad de Sevilla, Av. Manuel Siurot s/n, 41013 Seville, Spain; 2 Departamento de Fisiología Médica y Biofísica, Facultad de Medicina, Universidad de Sevilla, 41009 Seville, Spain; 3 Física Teórica, Universidad de Sevilla, Apartado de Correos 1065, 41080 Seville, Spain; 4 Facultad de Medicina, Departamento de Ciencias Fisiológicas, Laboratorio de Biomembranas, Universidad de Buenos Aires- CONICET, Instituto de Fisiología y Biofísica ‘‘Bernardo Houssay’’ (IFIBIO-HOUSSAY), Buenos Aires, Argentina

**Keywords:** Brain, Aqueduct, Ependyma, Cilia, AQP4^−/−^, Microarray, Planar cell polarity, CSF, CD11c, Microglia, Electron microscopy, Hydrocephalus

## Abstract

AQP4 is expressed in the endfeet membranes of subpial and perivascular astrocytes and in the ependymal cells that line the ventricular system. The sporadic appearance of obstructive congenital hydrocephalus (OCHC) has been observed in the offspring of AQP4^−/−^ mice (KO) due to stenosis of Silvio’s aqueduct. Here, we explore whether the lack of AQP4 expression leads to abnormal development of ependymal cells in the aqueduct of mice. We compared periaqueductal samples from wild-type and KO mice. The microarray-based transcriptome analysis reflected a large number of genes with differential expression (809). Gene sets (GS) associated with ependymal development, ciliary function and the immune system were specially modified qPCR confirmed reduced expression in the KO mice genes: (i) coding for transcription factors for ependymal differentiation (Rfx4 and FoxJ1), (ii) involved in the constitution of the central apparatus of the axoneme (Spag16 and Hydin), (iii) associated with ciliary assembly (Cfap43, Cfap69 and Ccdc170), and (iv) involved in intercellular junction complexes of the ependyma (Cdhr4). By contrast, genes such as Spp1, Gpnmb, Itgax, and Cd68, associated with a Cd11c-positive microglial population, were overexpressed in the KO mice. Electron microscopy and Immunofluorescence of vimentin and γ-tubulin revealed a disorganized ependyma in the KO mice, with changes in the intercellular complex union, unevenly orientated cilia, and variations in the planar cell polarity of the apical membrane. These structural alterations translate into reduced cilia beat frequency, which might alter cerebrospinal fluid movement. The presence of CD11c + microglia cells in the periaqueductal zone of mice during the first postnatal week is a novel finding. In AQP4^−/−^ mice, these cells remain present around the aqueduct for an extended period, showing peak expression at P11. We propose that these cells play an important role in the normal development of the ependyma and that their overexpression in KO mice is crucial to reduce ependyma abnormalities that could otherwise contribute to the development of obstructive hydrocephalus.

**Supplementary Information:**

The online version contains supplementary material available at 10.1186/s12987-024-00548-2.

## Background

Aquaporin 4 (AQP4) is the most important aquaporin in the brain and is expressed in the glial membranes, with the highest density over the endfeet membranes of subpial and perivascular astrocytes. AQP4 is also abundantly expressed in the ependymal cells that line the ventricular system [1, 2]. In recent years, numerous studies have demonstrated a prominent role for AQP4 in cerebrospinal fluid production (CSF) and circulation [3–5]. AQP4 is currently considered a major component of the glymphatic system, facilitating CSF flow through the brain parenchyma [4, 6]. In addition to serving as a water channel that facilitates the efficient movement of fluid between the various compartments present in the brain (blood, interstitium, intracellular space, and CSF), AQP4 constitutes an integral protein of the cell membrane in astrocytes and ependyma [1, 7] and provides structural support in both cases to their cellular membranes. In pericapillary astrocytes, AQP4 is a membrane component that participates in the structural assembly between astrocytes and the extracellular matrix surrounding endothelial cells [8]. AQP4 anchors to the cell membrane in the astrocyte endfeet by co-expression with dystroglycan and binds intracellularly with α-syntrophin, α-dystrophin and dystrobrevin and extracellularly with laminin and agrin [9, 10], as part of a protein scaffold collectively called the dystroglycan complex [11, 12], a structural component of the blood–brain barrier [4, 13]. In ependyma, AQP4 is associated with connexin 43 at the gap junctions on the basolateral membranes between adjacent ependymocytes, stabilising the cell–cell interaction between these cells and maintaining ependymal integrity in adult mice [14].

Abnormal AQP4 expression or distribution has been associated with hydrocephalus [13, 15], a pathophysiological condition of the central nervous system (CNS) that consists of an abnormal accumulation of CSF in the intraventricular cavity. Hydrocephalus occurs in foetal and adult stages and is associated with numerous variants and certain syndromes [16, 17]. The origin of hydrocephalus can be congenital or acquired; the most common congenital type would be structural anomalies of the brain such as neural tube defects, agenesis of the corpus callosum and cerebral aqueduct stenosis, while the acquired types are usually secondary to causes such as tumours, ventricular haemorrhages, CNS infection, and head trauma [17–21].

The aetiology of childhood hydrocephalus has been reported as prenatal in 55% and perinatal in 44% of children with this disease. Spina bifida, aqueductal stenosis, and low-weight preterm birth with ventricular haemorrhaging have been reported as among the most common causes of hydrocephalus [17, 19]. In general, hydrocephalus can be communicating, that is, without obstructions in the ventricular communications, or obstructive, if CSF flow is blocked in the ventricular system.

Radial glial cells originally function as stem cells, giving rise to specific cell types of the CNS, including astrocytes and ependymal cells that essentially constitute the membranes of the CNS/CSF interface [22]. Ependymal cells are specialised epithelial cells that constitute a monolayer of multiciliated cells with a cuboidal or columnar structure, also known as ependyma, that line the walls of the ventricular cavities. Given their cellular characteristics and their location in a border zone between the brain parenchyma and the CSF, these epithelial cells play relevant functions in CNS physiology and, especially, in CSF homeostasis: propulsion of ventricular CSF, transport between the brain parenchyma and CSF, and protection against substances. For their development, ependymal cells have multiple mechanisms that regulate their conversion from an immature cell at birth (P0) to a differentiated functional cell after the first postnatal weeks (21st day postpartum, P21). Their maturation process involves the development of multiple structures, among them the presence of cilia and the acquisition of their normal morphology, structure, and function, giving rise to translational and rotational planar cell polarity (PCP) [23, 24]. Mutations in the structural genes of the cilium and/or genes that regulate how cilia are distributed with a specific order and pattern on the surface of the ependymal cell give rise to ciliopathies, which can sometimes give rise to hydrocephalus [25].

Defects in the proliferation and differentiation of adult stem cells derived from the subventricular zone and genetic alterations that produce abnormalities in the ependyma have been demonstrated in AQP4-KO-CD1 (AQP4^−/−^) mice [14]. Various animal models, including hop gait mice with congenital hydrocephalus [26–28] and hydrocephalus produced by injecting kaolin into the cisterna magna of rats [27, 28], have demonstrated an association between hydrocephalus and AQP4 expression. Changes in AQP4 expression and cell–cell binding proteins (such as N-cadherin and Connexin 43) at the CNS/CSF interface have been associated with hydrocephalus in humans and animal models [14, 29]. Consistent with this association, Sylvian aqueduct stenosis occurs in 10% of AQP4^−/−^ mice offspring, causing death during the first month of life due to the onset of obstructive congenital hydrocephalus [30].

The present study therefore analyses the role of AQP4 in the formation and development of the aqueductal ependymal zone, using AQP4^−/−^ (AQP4-KO/C57BL/6) mice that lack full AQP4 expression, as well as control or wild-type (WT) mice. The study focussed on the mice at 11 days after birth (P11), given that in the AQP4^−/−^(AQP4-KO/CD1) mice used by Feng et al. [30], the sporadic hydrocephalus phenotype was observed starting at this stage of development and because the highest AQP4 mRNA expression was observed in the periaqueductal zone of WT mice at that age, as shown in the present work. Our study addresses the genes affected in their expression due to the absence of AQP4; and from the functional experiments, we hypothesised how the genes could contribute to the onset of hydrocephalus in the progeny of the AQP4^−/−^ (AQP4-KO/CD1) mice. In the AQP4/C56BL/6 mice we did not observed hydrocephalus at any age and the ventricles in these animals were smaller than the age-matched controls in all age ranges that we observed. Moreover, this study describes, for the first time, the expression of a microglial subtype, CD11c + , which plays a crucial role in the normal formation of aqueductal ependyma, which will remain highly expressed in the subependymal zone of the aqueduct for a longer time when the mice lack AQP4, indicating the crucial role of CD11c + for the development of this periaqueductal tissue.

## Materials and methods

### Animals

Wild-type C57BL/6 male and female mice (WT), or with deletion of the gene encoding AQP4 (AQP4^−/−^/ C57BL/6) were housed in a regulated temperature environment (22 ± 1 °C) in a 12 h light/dark cycle, with ad libitum access to food and water. The AQP4^−/−^ mice were genotyped as indicated previously [31]. Mice were sacrificed under deep anaesthesia induced by a combination of 100 mg/kg of ketamine (Pfizer) and 10 mg/kg of xylazine (Bayer). All experiments were carried out according to the European Directive 2010/63/EU and the Spanish RD/53/2013 on the protection of animals used for scientific purposes. Animal procedures were approved by the Animal Research Committee of the Virgen del Rocío University Hospital (26/01/2017/017; University of Seville).

### RNA extraction and quantitative reverse transcription PCR analysis

Cerebral aqueducts from P11 animals, age 11 days after birth, were microdissected in ice cold diethyl pyrocarbonate (DEPC)-PBS under a stereoscopic binocular microscope (Olympus SZX16) from fresh brain coronal sections (thickness 1 mm). Total RNA was isolated using the RNeasy Micro Kit (Qiagen) according to the manufacturer's instructions. cDNA synthesis was performed using the QuantiTect reverse transcription kit (Qiagen) and relative mRNA expression levels were measured using quantitative real-time polymerase chain reaction (RT-qPCR) with the ViiA 7 real-time PCR system (Thermo Fisher). RNA expression levels were normalised using 18S ribosomal mRNA to correct for variations in RNA input amounts, and all samples were analysed in triplicate. Primer Express software v2.0 (Applied Biosystems) was used to design all primers used (Table 1). The quantity and purity of RNA and cDNA were assessed with a NanoDrop ND-1000 UV–vis spectrophotometer (Thermo Fisher).

**Table 1:** Primers for the respective genes employed in the study

Gene	Forward primer	Reverse primer
FoxJ1	CCGCCACAACCTGTCCTT	GGTCGATGCGCCAGAAAC
Rfx4	TCCCCCATCGAGTCTTACATAGA	GCAAGACCACATCAGCAGGAA
Rfx3	TCTATCACCTACCCCCTCTGTCA	TGTCTGAACCCGTCTCTGAAGTC
Enkur	CCCAAGCCGATTTATGTTGAC	ACATCCTCATTTCGCTTGCAT
Ift122	AGACAAAGCGGAGCAGTGTATCTA	CAGAGGTGTCATAAACCAGCAATC
Dnah12	GCCAGGTTGTTCTTTGTGTGTCT	CCTCTCACCAGCTCCACAATG
Rsph9	GAGTATGAACACACAGAGTTGCAGAAG	TGATGGACACCAAGCGAGTCT
Spag16	TCGGAAGATGAATGCGAATATG	CTTGCTCTCCAATGATGTGAATTG
Hydin	TTCTGCTGGTCCGAAACATTG	TTCGCCCACGTTGAGAATC
Cfap43	AAGGACGGCATACTCGCTTCT	GTCACCGGCCCATCAAACT
Cfap69	AAGCCTATGGACCTTAATCGTATCA	TGTGCCAAATCTCTTAGAGGAAATC
CCdc170	TCCGCTCCAAGATGCTTTCTA	GAGACGTGAGGAGAGCTGACTTTC
Cdhr4	TTCCAGCATTCAGCTTCCATCT	AACCTCTGCTGCTCATAGTCCAA
Gja1	CAACCCGGTTGTGAAAATGTCT	AGCTTCTCTTCCTTTCTCATCACATAG
Gjb6	ACCACTACCGCTGCTGATGTC	GGTCCCCCAGTCCATCGT
Spp1	CAGCCAAGGACTAACTACGACCAT	AGCTGCCAGAATCAGTCACTTTC
Gpnmb	GTCTGGCACCTACTGTGTGAATTT	AGGACACCATTCACTGCTCTCA
Itgax	CAACTAGGTGACCTCCGAAGTACTG	AAGATGGCCCGGGTACTCA
Cd68	TGCGGCTCCCTGTGTGT	GAAGGATGGCAGGAGAGTAACG
P2ry12	GGCTCAGCCAGAAACATTGTAA	CCGGTGTCCTGGAAAGTGTTA
Tmem119	CGGCAGGCAGACAGTCAGA	TCGCAAGTAGCAGCAGAGACA
Sall1	CGTGAGCGGCTGATGTTTG	ACCCTTCTCTGTGTCCCCATCT
Igf1	CCACACTGACATGCCCAAGA	TCCTGCACTTCCTCTACTTGTGTT
Trem2	CCCGGAGGTACGTGAGAGAA	GAAGCAAAAGTAGCAGAAACAGAAGTC
18S	AACGAGACTCTGGCATGCTAA	GCCACTTGTCCCTCTAAGAAGT

### Microarray analysis

For the transcriptomic analysis of tissue samples, the ClariomS^TM^Micro Assay mouse kit (Thermo Scientific; Affymetrix) was used. A total of 3 samples per condition were included in the study (each one being a pool of 3 independent biological samples). The integrity of the RNA was evaluated by capillary electrophoresis with the 2100 bionalyzer (Agilent). The preamplification of the extracted RNA was carried out using the GeneChip^™^ IVT Pico kit (Thermo Scientific) and according to the protocol standardised by the manufacturer. Next, 5 μg of the resulting cDNA was fragmented and labelled for hybridisation in the ClariomTM Mouse Assay (Thermo Scientific). This was followed by the washing, labelling (GeneChip^™^ Fluidics Station 450; ThermoScientific) and scanning (GeneChip^™^ Scanner 3000; Thermo Scientific) steps, which were performed according to the provided protocols.

The data were preprocessed (with background correction, normalisation, and summarization) using the RMA (Robust Multichip Average) method, using the BrainArray annotation library pd.clariomsmouse.mm.entrezg (version 25.0.0) for mapping probes to genes. Differential expression analysis was performed with the limma package (version 3.46.0) [32]. To extract biological information from the list of differentially expressed genes, gene cluster enrichment analysis (GSEA) was performed [33]. The gene sets used come from the Signature Database (v7.4). Finally, through the TAC software (Transcriptome Analysis Console; Affymetrix, Thermo Scientific) we obtained the graphs of the Volcano-type analysis and the heatmap shown in Fig. 2.

### Comparative transcriptomic analysis of microglial populations

We access data provided by different studies that characterised the gene profiles of different microglial subtypes: PAM (Proliferative region-associated microglia [34]), ATM (Axon tract-associated microglia [35]), CD11c [36], APP (Microglia isolated from the brain of Alzheimer's animal model APPswe/PS1dE9 [37]), Aged (Microglia isolated from the brain of aged mice [38]), DAM1 and DAM2 (Disease Associated Microglia, type 1 and 2 [39]), MGnD (Neurodegenerative microglia, [40]), or LDAM (Lipid-droplet-accumulating Microglia, [41]). Then a comparative bioinformatic analysis was carried out based on clustering methods [42, 43]. In this analysis, the differential expression levels of each gene were defined as attributes that characterise a specific cell type. From this set a vector space was defined whose dimensionality was determined by the number of attributes (genes). For n attributes, each population was defined in this space by an n-dimensional vector, that is, v ≡ (v_1_, …, v_n_). Once the vectors for each population are defined, the similarity between cells is established using the Euclidean distance. If we have two populations, i and j, the distance between these populations, denoted as ${d}_{i,j}$ is calculated as follows:

$${d}_{i,j} \equiv \sqrt{\sum_{k=1}^{n}{\left({v}_{k}^{i}-{v}_{k}^{j}\right)}^{2},}$$

In this way, the smaller the distance between populations, the more similar they are to each other. We also determine the correlation function between two populations, i and j, ${C}_{i,j}$​, by projecting the vectors onto each other:

$${C}_{i,j}\equiv \mid {\text{cos}}\theta \mid = \frac{\mid {v}^{i}\cdot {v}^{j}\mid }{\mid \mid {v}^{i}\mid \mid \mid \mid {v}^{j}\mid \mid }$$

where ${v}^{i}\cdot {v}^{j}\equiv {\sum }_{k}{{v}_{k}^{i}v}_{k}^{k}$ is the Euclidean scalar product,

$\mid \mid {v}^{i}\mid \mid \equiv \sqrt{{\sum }_{k}{({v}_{k}^{i})}^{2}}$ is the magnitude,

and θ is the angle between the vectors.

Since $\mid {\text{cos}}\theta \mid \in \left[\mathrm{0,1})\right]$ the value of ${C}_{i,j}$ determines if there is any correlation between populations. A zero value indicates total independence (the vectors are perpendicular in the attribute space), which means there is no relationship between the compared populations. Conversely, a value of one is given when the populations are entirely equivalent (linearly dependent vectors). The obtained dot product values were represented in matrices (Python generated), indicating comparisons between different groups.

### Hybridization in situ (RNAscope)

To perform histological analyses, mice on postnatal day 11 (P11) were anaesthetised and intracardially perfused with 10 ml of buffered phosphate saline (PBS, Sigma) and 4% paraformaldehyde (PFA, Sigma) in PBS. Subsequently, the brains were immediately removed and fixed with 4% PFA in PBS for 2 h at 4 °C, cryoprotected in 30% sucrose in PBS at 4  C for 24 h and embedded in the optimal cutting temperature (OCT) compound (Tissue-Tek) prior to storage at − 80° C. 20 µm coronal brain slices were obtained with a cryostat (Leica) and placed on Superfrost Plus slides (Thermos Scientific) for their use. To visualise the aqueduct, slices were taken from the bregma coordinates − 4.48 and − 5.02 mm [44]. In situ hybridization by the RNAscope® technique was performed according to the manufacturer's instructions (Advanced Cell Diagnostics (ACD) for fixed frozen tissue sections as previously reported [44]. Antigen retrieval and protease treatment were carried out according to the kit instructions (RNAscope H2O2 & Protease Plus ACD #322,330). The Spp1 probe (ACD #435,191) was hybridised for 2 h followed by 6 amplification steps, using a HybEZ oven (ACD). The signal was detected with the Brown RNAscope 2.5 HD detection kit (ACD #322,310), with an incubation time of 10 min. Slices were mounted with Fluoromount-G mounting medium (Invitrogen) and analysed in wide-field microscopy with a Leica DMi8 inverted microscope with THUNDER computational clearing software.

### Immunofluorescence

Coronal brain Sects. (30 μm thick) were cut in a cryostat (Leica) and mounted on Superfrost Plus slides (Thermos Scientific). The IF protocol lasts two days and details were previously described [45] and briefly summarized as follow. The slices were washed twice for 5 min in 0.1 M PBS, then permeabilized with the application of PBT-0.3% [0.1 M PBS with 0.3% (v/v) Triton (Sigma)] and washed with PBT-0.1% on two occasions during 5 min before the blocking step. This step was carried out in 200 μl of blocking solution [10% goat or horse serum (Sigma) and 1 mg/ml bovine serum albumin (BSA, Sigma), in PBT-0.1%], using incubation chambers (Electron Microscopy Sciences). After 1 h, the solution was removed and the different primary monoclonal antibodies used anti-vimentin (1:500; Abcam), anti γ-tubulin (1:1000, Sigma), anti Osteopontin (Spp1, 1:100, Santa Cruz), anti CD11c (1:500, Bio-Rad) and anti Iba1 (1:5000, Wako), were dissolved in blocking solution at the specific concentrations and left for incubation overnight at 4 ºC. Anti-mouse IgG conjugated with Alexa Fluor488 (1:400, Invitrogen), anti-rabbit IgG conjugated with Cyanine CyTM3 (1:200, Jackson Immunoresearch), anti-Hamster Armenian IgG conjugated with Alexa Fluor488 (1:400, Abcam) and anti-mouse conjugated with Alexa Flour653 (1:400, Invitrogen) were used as secondary antibodies, for two hours in the dark. The nuclei were stained with DAPI (1:1000, Sigma). The tissue sections were mounted with Dako fluorescence mounting medium (Dako). Confocal images were acquired using a Leica Stellaris 8 confocal microscope. In all cases, defined areas were established in the histological section and several layers were acquired reading on the Z axis (Z-stack), defining a distance of 0.7 μm between the layer and layer. The images obtained were evaluated with FIJI [46] and in certain cases three-dimensional reconstructions of the obtained markings were performed using Imaris Software (Bitplane, Belfast, United Kingdom) for this purpose.

### Quantification of apical area in ependyma cells and evaluation of ciliary basal body orientation

The apical ependymal surface was measured by processing vimentin immunolabeling micrographs and using FIJI software. At least 30 cells per image were quantified. Five to six ependymal regions per animal were subjected to analysis, and the mean values per individual were represented. These images were also used to evaluate the orientation of the basal body following a methodology based on FIJI and described by different authors [47, 48]. First, the cells were delimited by their vimentin labelling and the cell centre was assigned. Then, the central point of the basal bodies or the centroid was located by γ-tubulin signal. Finally, we generate vectors that point from the centre of each cell to its centroid. Data were processed and analysed using R software (http://www.R-project.org/) and Polar histograms were designed with Python software through the Seaborn library [49].

### Confocal microscopy ex vivo

To assess ciliary mechanical activity, we recorded their activity through ex vivo microscopy observations following an adaptation of the methodology described by other authors [50]. Brains from sacrificed animals were removed without prior perfusion and quickly processed into 1 mm sections using a matrix. The coronal section containing the cerebral aqueduct region of interest was washed in 0.1 M PBS at 37  °C and transferred to a glass bottom dish containing high glucose DMEM. Ciliary activity was recorded in this medium, maintaining a temperature of 37  °C and a gas mixture of 95%/5% O2/CO2. Due to the high speed required for image capture (at a rate of 60 frames per second), the resonance scanning mode was employed. For this approach, a 25 × water immersion objective was used and image analysis was conducted using FIJI software, defining regions of interest (ROIs) in the images containing ciliary components with periodic movement. For analysis, strategies developed by other researchers [51, 52] were employed, which, in summary, involved obtaining kymograph-type images to determine the beat frequency per second. Between 3 and 5 ROIs were examined for each area, and a total of 5 images per animal were analysed, with mean frequency values represented in the results obtained.

### Electron microscopy

For electron microscopy, mice were perfused transcardially with 2.5% glutaraldehyde in phosphate or cacodylate buffer (0.1 M, pH 7.5), and the brains were postfixed for 2 h in the same fixative. For scanning electron microscopy (SEM), 300 μm-thick sagittal brain slices containing the cerebral aqueduct region were obtained with a vibratome (Leica VT1000M), dehydrated to a critical point, and prepared for observation using a Zeiss Evo environmental scanning electron microscope (Zeiss, Oberkochen, Germany). A minimum of 10 images per sample and 3 samples per condition were evaluated. For transmission electronic microscopy (TEM), a dissection of the anatomically region of interest was performed after vibratome cutting. Subsequently, these samples were rinsed, post-fixed in 2% osmium tetroxide, and embedded in epoxy resin. Ultrathin sections from the samples were obtained and examined with an electron microscope (Zeiss Libra 120).

### Analysis of rotational polarity

To estimate rotational polarity, images were taken at 12,500 magnifications, revealing clusters of cilia ranging from 10 to 15 in number per image. Then, FIJI software was employed to obtain the orientation of the central pair of microtubules, and each individual rotational deviation was estimated in relation to the average angle of the grouped cilia of the image. No less than 10 images and 100 cilia per mouse were considered for the analysis.

### Immunogold

Preembedding immunogold staining was performed with vibratome sections. First, the sections were washed in PBS and incubated in a 50 mM glycine solution for more than 10 min. After the standard procedure, the samples were incubated with polyclonal anti-AQP4 (1:50; Alomone) or anti-IBA1 (1:50; Wako) at 4 °C overnight. The sections were then washed in PBS and an incubation with 1.4 nm gold-AF488 conjugated secondary antibody (1:25, Nanoprobes) was applied for 2 h at room temperature. Subsequently, the samples were further fixed in a PBS buffer containing 1% glutaraldehyde at room temperature and treated with enhancer reagents (Goldenhance^™^ EM Formulation, Nanoprobes) for 5 min to boost the signal. In antibodies-labelled final step, the sections were processed following the method we described earlier, including osmium fixation, dehydration, and embedding procedures. Negative control experiments were conducted without the primary antibody.

### Statistical analysis

Data from experiments are presented as mean ± standard error of the mean (s.e.m.), and the statistical test performed is specified in each figure legend. For all the analyses performed, the data were tested for normality using the D’Agostino and Pearson test or the Shapiro–Wilk test. When these properties were confirmed, variance analysis was performed with Tukey's HSD post hoc analysis for multiple group comparisons or Student's t test (for two-group comparisons); otherwise, the nonparametric Kruskal–Wallis H test (for multiple comparisons) or Mann–Whitney U test (for two-group comparisons) was used. The analysis of the distributions for circular data (study of translational polarity) was carried out with the Watson homogeneity test for two samples (U2 test). This was carried out in the R statistical programme, using a free code provided by other authors [53]. All statistical analyses and graphs were performed with the GraphPad Prism 8.4.2 version.

## Results

### Time course of AQP4 expression in the periaqueductal zone during brain neurodevelopment

**Fig. 1:**
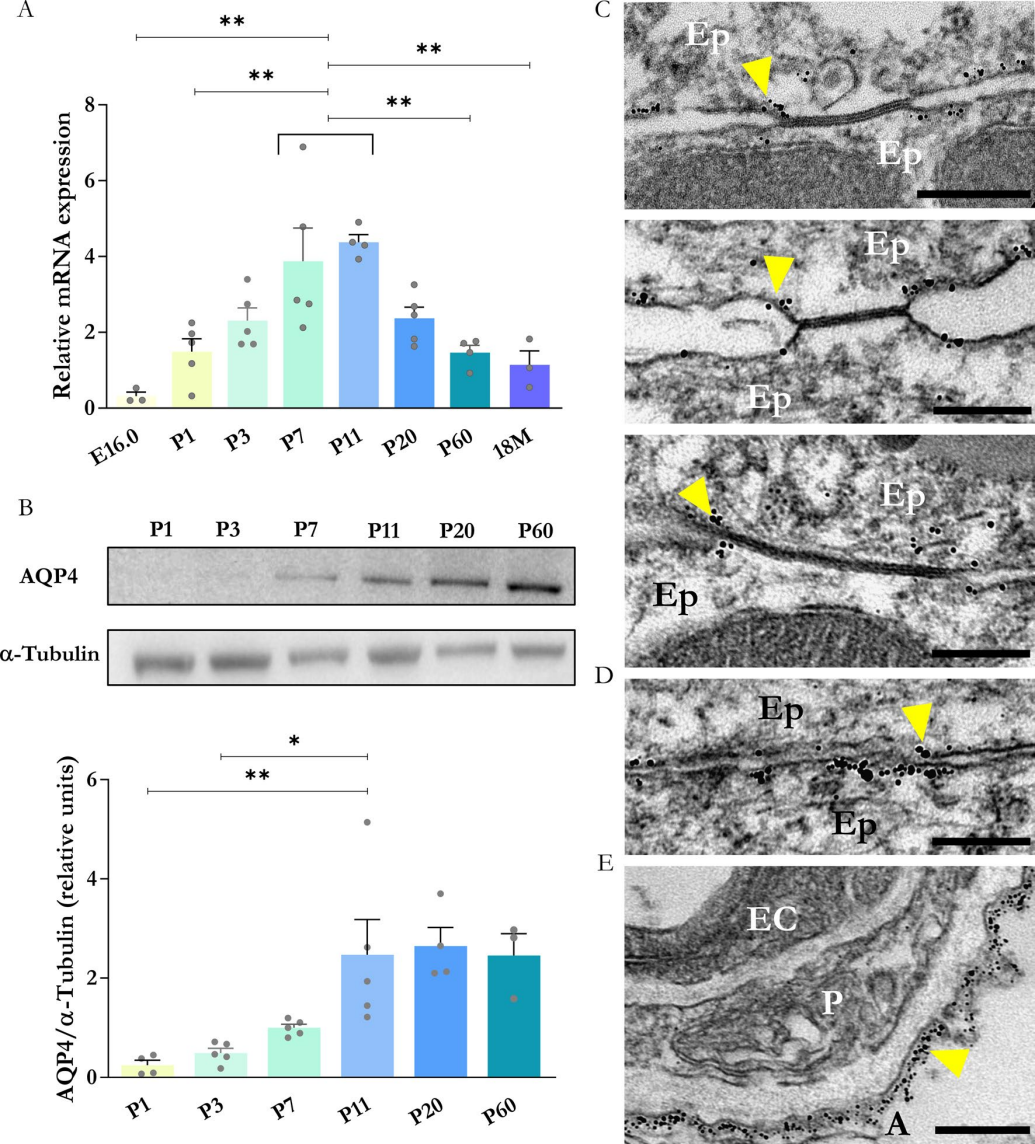
Temporal expression pattern of embryonic and postnatal molecular AQP4 expression in the cerebral aqueduct ependyma. **A** Changes in relative gene expression of *Aqp4* (determined by RT-qPCR) in the periaqueductal tissue during developmental stages from E16.0 to 18 month-old mice (18 M). N = 3–5 samples per condition. **B** Quantification of relative AQP4 protein abundance by western blot in the periaqueductal tissue of the mice between stages P1 and P60. N = 3–5 samples per condition. Data are presented as mean ± SEM. Significant differences between groups were assessed using one-way ANOVA followed by Tukey’s post hoc test (*p < 0.05, **p < 0.01). **C** Transmission electron microscopy micrographs showing AQP4 immunogold labelling in junction regions between ependymal cells **C** and **D** and in astrocytic endfeet facing the perivascular space **E**. Yellow arrowheads indicate colloidal gold particles identifying the presence of AQP4. *Ep*  ependymal cell, *EC*  Endothelial cell, *A*  Astrocytic endfeet, *P*  Pericyte. Scale bar = 200 nm

We evaluated the abundance of AQP4 in the periaqueductal zone of WT mice by quantifying the levels of gene and protein expression by RT-qPCR and western blot, respectively. These analyses included tissues extracted at age E16 and during the neonatal period (P1-P7, previously studied [45]) and were extended to later postnatal points (P11 and P20), as well as tissues from adult (P60) and older (18 months old [18 M]) mice. As shown in Fig. 1A, mRNA levels showed a significant increase from E16 to P11 and, after that point, a gradual but also significant decrease in AQP4 mRNA levels, such that at 18 M, levels were similar to those seen at P1. The protein expression levels (Fig. 1B) showed very low values at the earlier postnatal ages (P1-P3) and then significant increases between P7 and P11, remaining stable in later stages of adulthood. To characterise the protein’s location in the tissue observed in P11, immunogold labelling was performed, and transmission electron microscopy (TEM) images helped locate AQP4 at the ultrastructural level in the ependymal cells of the aqueduct (Fig. 1C–E). Colloid gold particle accumulation can be observed on the basolateral membrane of ependymal cells (Fig. 1C and D). Labelling appeared to show a preferential tendency for areas close to intercellular gap junction spaces (Fig. 1C, yellow arrowhead), as well as in regions with a higher density of cytoskeleton fibres as adherent-type junctions (Fig. 1D, yellow arrowhead). A high density of gold particles was observed in the astrocyte feet, which delineate the perivascular space. This density was greater than that observed in the ependymal membrane (Fig. 1E). The following sections compare the brain samples obtained from the WT mice with AQP4^−/−^ at P11 to better understand the relevance of AQP4 for neurodevelopment. At this stage, the highest expression of AQP4 mRNA was observed in the periaqueductal zone of the WT mice.

### Transcriptomic analysis by microarray of ependymal development in the cerebral aqueduct

**Fig. 2:**
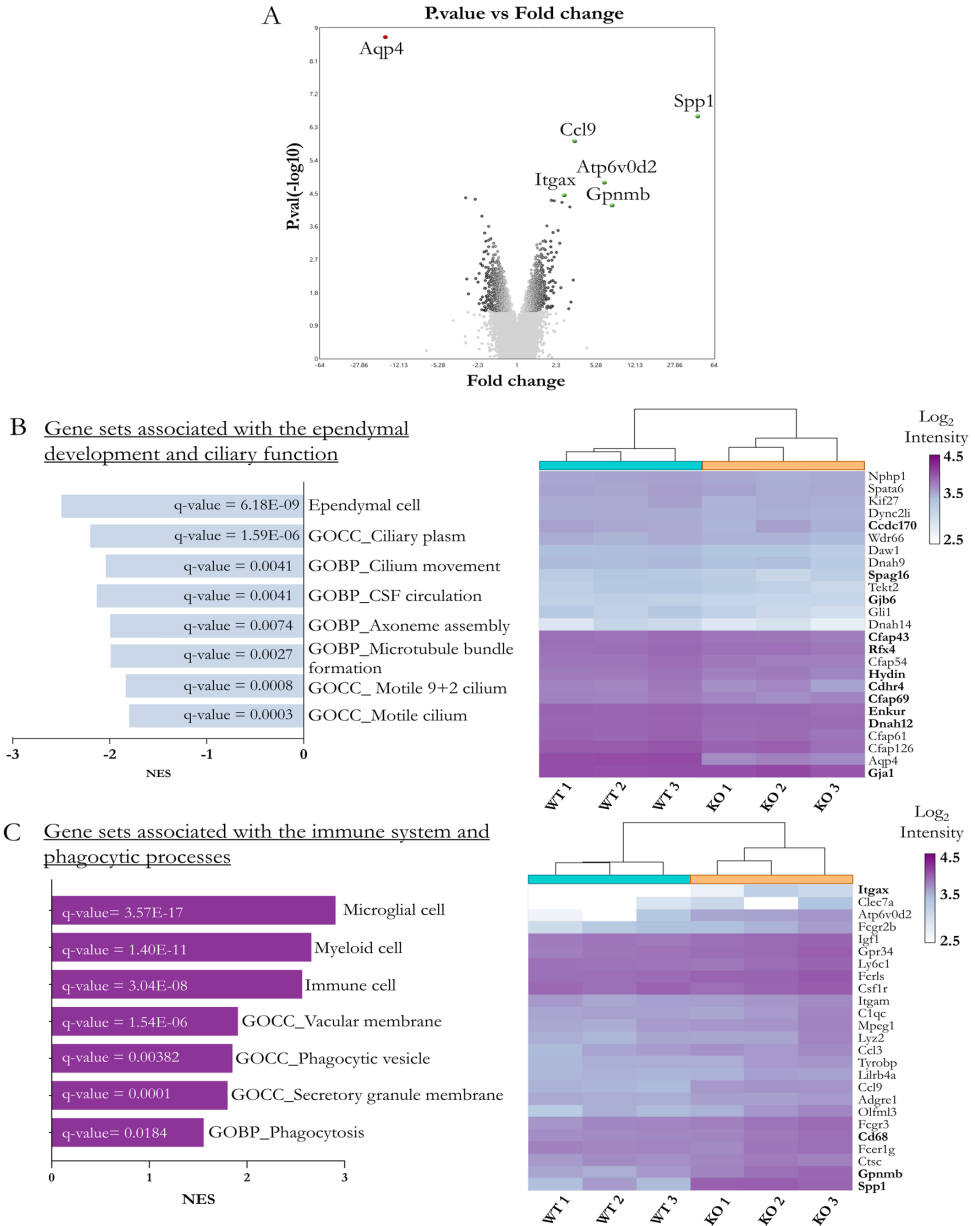
Microarray-based comparative transcriptome analysis of the cerebral aqueduct ependyma of WT and AQP4^−/−^ mice at P11. **A** Volcano plot detailing genes significantly expressed by FDR < 0.05 criteria (green indicates overexpression, red indicates underexpression, given the KO/WT comparison). The 809 genes that exceeded the statistical criteria of p < 0.05 are indicated in dark gray. **B**–**C** The plots reflect gene sets (GS) associated with ependymal **B** and immune functions **C** that showed differential expression. On the right side of each heatmaps, significant genes belonging to specific gene enrichment pathways (GS) are shown. In bold are indicated those genes validated by RT-qPCR

When comparing the AQP4^−/−^ mice versus the WT mice at P11, 809 genes (431 down and 378 up) were identified to have altered expression in the cerebral aqueduct. In the volcano plot (Fig. 2A), the points identify the genes that met the statistical criteria of p < 0.05.

As shown in Fig. 2B, the analysis showed a decrease in gene enrichment of gene sets (GS) associated with the ependymal population (normalised enrichment score [NES] of − 2.50) in the AQP4^−/−^ mice. There was also a decrease in the expression levels of genes grouped under ontological terms (GO) of biological processes related to CSF circulation and cilia assembly and in the GO of the cellular components referring to the structure of the cilia.

The tissue from the KO mice showed extensive enrichment in the gene profile of the immune populations (Fig. 2C). Specifically, greater overexpression was identified for genes associated with a microglial population and myeloid cells (NES of 2.90 and 2.66, respectively). Similarly, the GO analysis highlighted significant increases in functions such as phagocytosis and in components such as the vacuole membrane and secretory granules. In both heat maps shown on the right side of each comparison (Fig. 2B and C), significant genes are indicated, and those genes that were validated by RT-qPCR are in bold.

### 3- Validation of the transcriptomic analysis results of genes related to the ependyma cells and their cilia

**Fig. 3:**
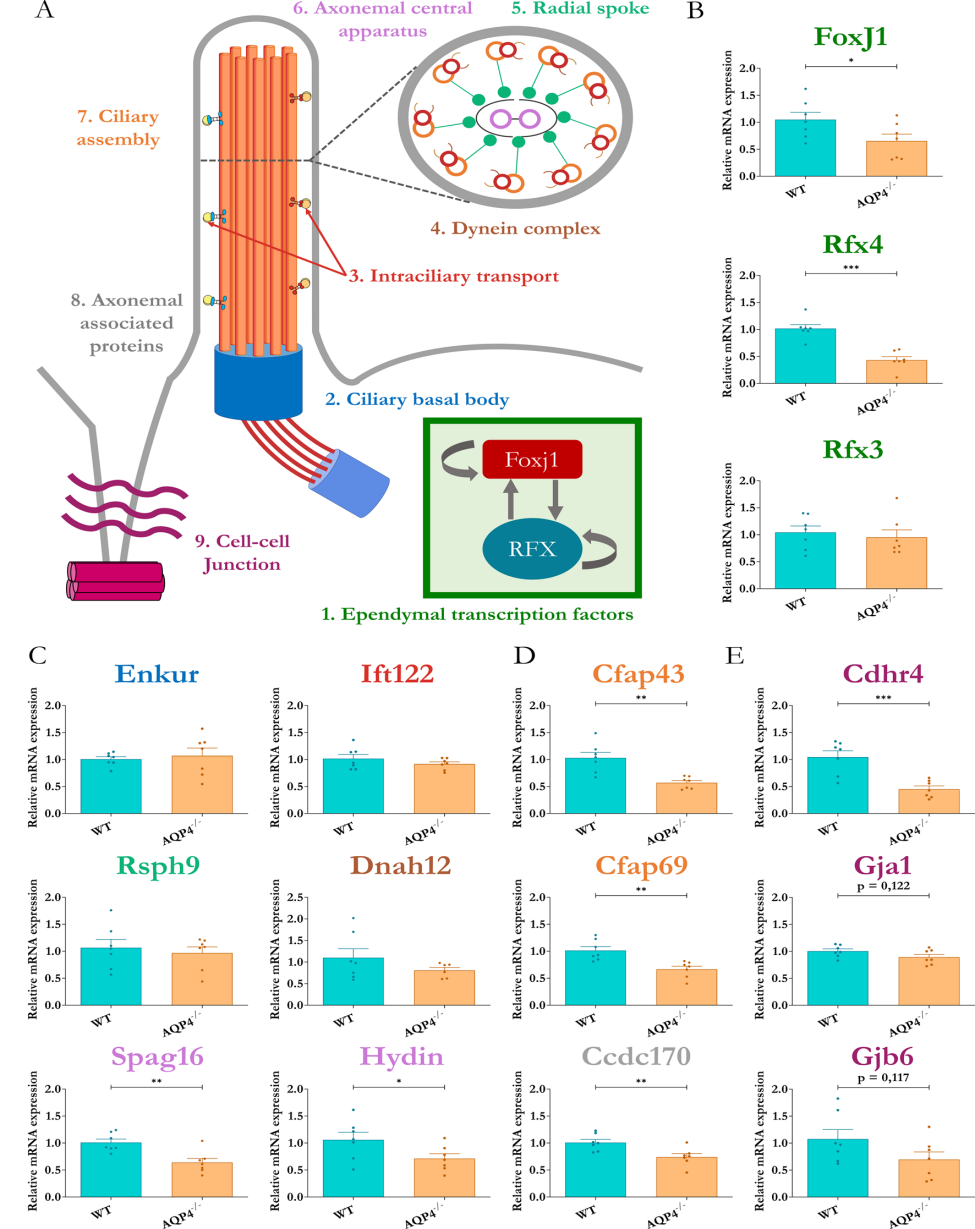
Analysis of differential gene expression involved in ependymal differentiation and ciliary maturation. **A** Schematic representation of gene classification according to the ciliary/ependymal component they encode for (inspired by Patir et al. 2020). **B**–**E** Gene expression quantification by RT-qPCR for the genes related to ependymal development (colour-coded) in cerebral aqueduct ependyma of WT and AQP4^−/−^ mice at P11. Comparison of expression levels in genes associated with ependymal transcription factors, FoxJ1, Rfx4 and Rfx3 **B**, axonemal components and cilium formation, Enkur, Ift122, Rsph9, Dnah12, Spag16 and Hydin **C**, axonemal constitution, Cfap43, Cfap69, Ccdc170 **D**, and junction complexes, Cdhr4, Gja1, Gjb6 **E**. N = 7 per condition. Unpaired Student’s t-test employed for statistical significance analysis. *p < 0.05; **p < 0.01; ***p < 0.001

The differential gene expression observed in the transcriptomic study for various genes was validated by qPCR. These genes were grouped according to the function of the protein they encode (Fig. 3A), with the objective of determining whether the defect occurs in a generalised manner or is specific to certain parts of the structure to which they belong. Nine groups were formed with the selected genes: (i) transcription factors, (ii) cilia basal body, (iii) intraflagellar transport, (iv) dyneins, (v) radial spines, (vi) central apparatus, (vii) cilia assembly, (viii) associated proteins, and (ix) cell–cell junctions. Notice the different colour code for different gene sets in Fig. 3A. Following this scheme, we observed significant decreases in the KO mice in the expression of genes encoding transcription factors Rfx4 (***p < 0.001) and FoxJ1 (*p < 0.05), determining factors for ependymal differentiation that, together with Rfx3 (without significant changes), mark the beginning of ciliogenesis (Fig. 3B). As for Rfx3, no significant changes were observed for the genes that encode the basal body protein (Enkur), the intraflagellar transport protein (Ift122), the cytoskeletal motor protein (Dnah12) or the radial spine protein (Rsph9) (Fig. 3C). However, a significant reduction in the expression of genes involved in the constitution of the central apparatus of the axoneme such as Spag16 (**p < 0.01) and Hydin (*p < 0.05) was also observed in the AQP4^−/−^ mice (Fig. 3C). Decreased expression of Cfap43 and Cfap69 (**p < 0.01), both of which contribute to cilia assembly, and of Ccdc170 (**p < 0.01), a gene related to associated proteins relevant for support functions, was also detected in tissue from the AQP4^−/−^ mice (Fig. 3D). The expression levels of genes associated with intercellular junction complexes (Fig. 3E), such as Cdhr4 (***p < 0.001), encoding for cadherin-related family member 4, a junction protein of the adherens junction complex, showed a significant reduction. Although not significant, decreases were observed in the levels of Gja1 (p = 0.122) and Gjb6 (p = 0.117) transcripts that encode connexins 43 and 30, respectively. The validation of the results achieved by the transcriptome approach led us to investigate how the postnatal development of aqueduct ependyma is altered by the absence of aquaporin expression.

### Comparative analysis of PCP grade in aqueductal ciliated ependymal cells

Acquisition of planar cell polarity (PCP) is one of the most determinant processes in the development of ciliated ependymal cells and occurs during the postnatal phase. In this study, tests were conducted to assess PCP in its two components: translational polarity (organisation of the basal bodies of the cilia on the apical ependymal surface) and rotational polarity (acquisition of a homogeneous orientation of the cilia in the tissue).

### Translational PCP

**Fig. 4:**
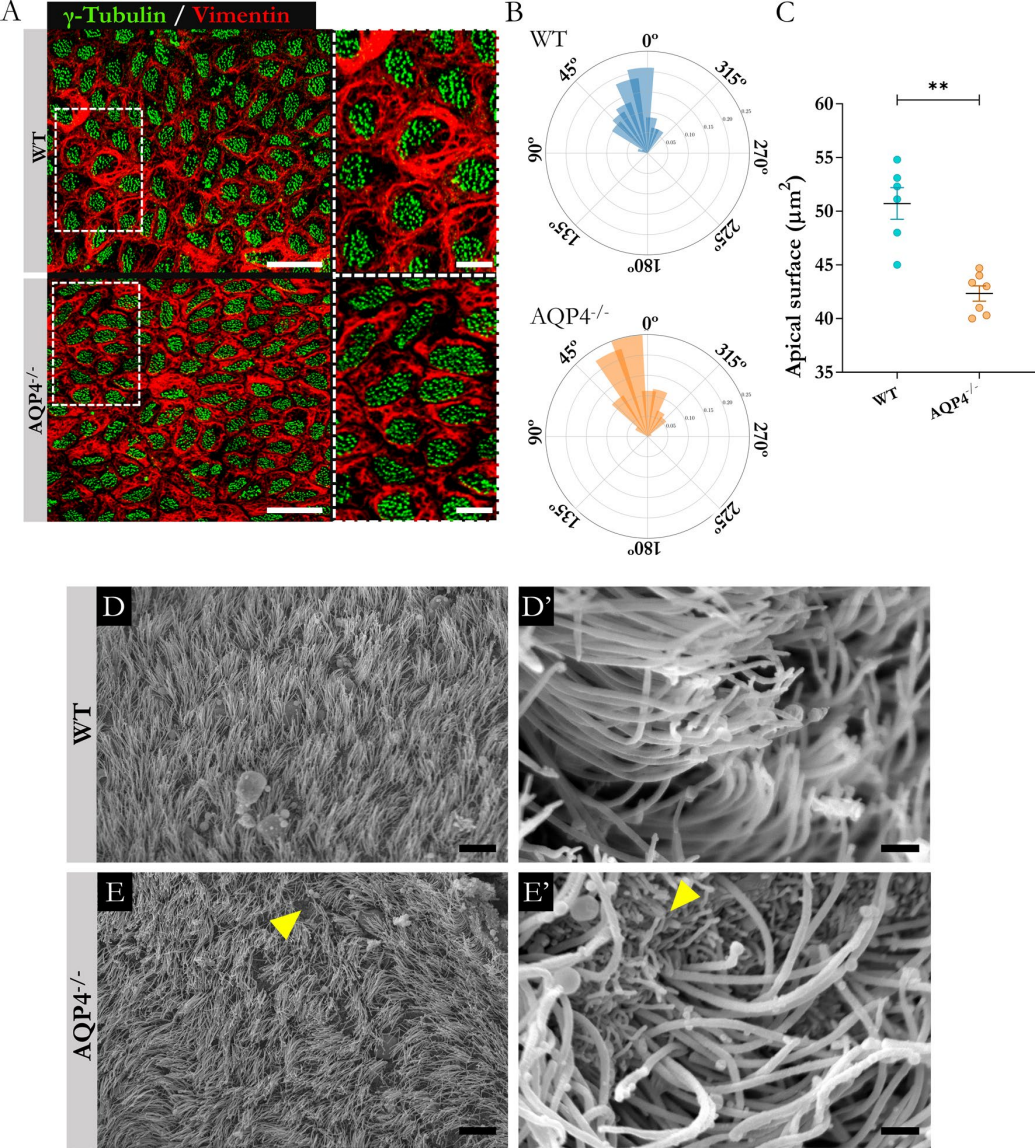
Translational polarity analysis and examination of ependymal cilia of the cerebral aqueduct. **A** Immunofluorescence labelling of cytoskeleton (vimentin) and basal bodies (γ-tubulin) in ependymal cells of the WT and AQP4^−/−^ mice at P11. The right side shows the area outlined in a higher magnification image. Scale bars = 15 µm in image and 5 µm in the insert. **B** Polar histograms representing the angular distribution of vectors of basal bodies around the mean in both conditions. **C** Quantification of apical area of ependymal cells of WT and AQP4^−/−^ mice (determined by vimentin signal). N = 6–7 per condition. Data are presented as mean ± SEM. Unpaired Student’s t-test employed for statistical significance analysis. **p < 0.01. **D**–**E’** Scanning electron microscopy evaluation of ciliary distribution on ependymal surface of the cerebral aqueduct. Scanning electron micrographs of the ependymal surface at the cerebral aqueduct level showing the orientation of ciliary structures identified in WT **D** and AQP4^−/−^ **E** mice at P11 (N = 3 and 4, respectively). As showed by the greater magnification images (**D**’–**E**’), greater homogeneity in cilia arrangement is exhibited in the WT condition. Yellow arrowheads reflect regions lacking cilia. Scale bars = 10 µm **D** and **E** and 1 µm (**D**’ and **E’**)

To determine whether there were translational PCP abnormalities in the ependymal cells of the AQP4^−/−^ aqueduct at P11, immunofluorescence assays were performed against the markers vimentin (intermediate cytoskeletal fibre used to delimit the cellular structure) and γ-tubulin (basal ciliary body marker) in brain sections containing the region of interest. From the images (Fig. 4A), the relative positions of the basal bodies were evaluated by creating vectors directed from the centre of the apical surface of the cells to the centre of the cilia groups (the procedure is described in greater detail in Materials and Methods). The orientations of the vectors obtained are represented in the polar histograms of Fig. 4B, which reflect significantly greater angular homogeneity (p < 0.001 Watson U^2^ test) for the WT ependymal cells (more than 75% in the interval between 0º-45º), compared with the greater dispersion for the vectors made in the transgenic tissue (only 50% for the same interval in the AQP4^−/−^ mice). Quantification of the apical area of ependymal cells was performed using vimentin-delimited labelling. The measurements showed a significant decrease (10%) in the area of the ependymal cells of the AQP4^−/−^ mice (Fig. 4C), a finding that could help explain the poorer translational PCP observed in the KO mice, given that a smaller surface for anchoring of the basal bodies would affect the location and orientation of the bodies in the membrane.

To obtain a high-resolution panoramic view of the ciliary organisation in the epithelium, scanning electron microscopy (SEM) observations of the aqueductal ependymal surface were performed in the WT and AQP4^−/−^ mice at P11. From the micrographs (Fig. 4D and 4E), we verified that both tissues shared the nature of the multiciliated epithelium; however, the arrangement of the ciliary structures varied depending on the condition. Thus, if we consider the WT mice (Fig. 4D), we can see how the cilia are homogeneously grouped in tufts (with cilia of surrounding cells pointing in the same direction). On the other hand, the ciliary tufts of the AQP4^−/−^ mice showed more irregular patterns (Fig. 4E), with heterogeneous orientation even in adjacent cells. Similarly, more areas devoid of cilia were observed that exposed their apical microvilli directly to the aqueductal space (yellow arrows). These observations, also highlighted at higher magnifications (Figs. 4D' and E'), were indicators of lower or poorer cilia orientation of the AQP4^−/−^ ependyma.

### Rotational PCP

**Fig. 5:**
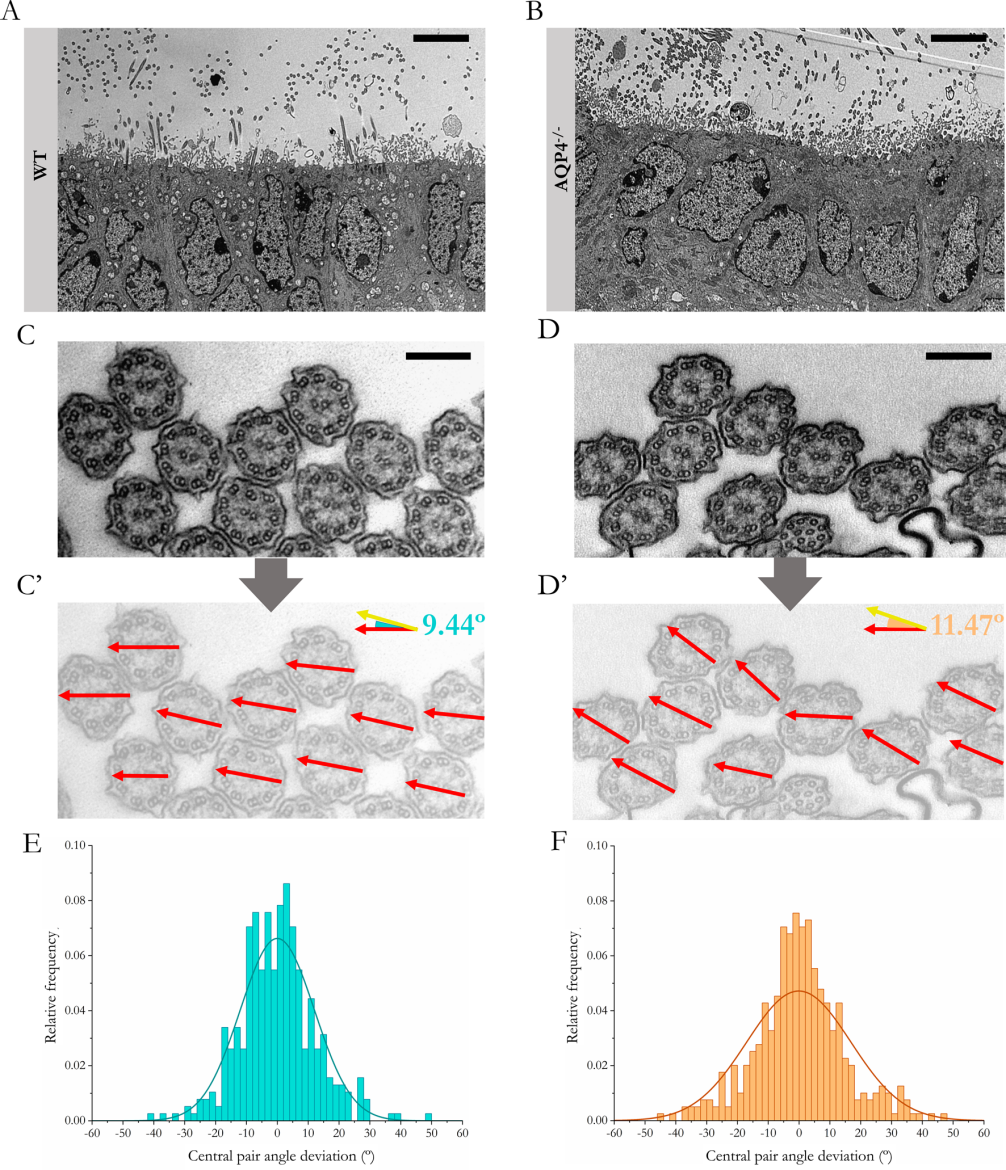
TEM evaluation of rotational polarity of ependymal cilia in the cerebral aqueduct. Representative micrographs showing the ultrastructure of ependyma bordering the cerebral aqueduct in the WT **A** and AQP4^−/−^ **B** mice at P11. Study of the rotation angle of the central pair of microtubules in cross-sections of ciliary axonemes exposed to the aqueduct in WT (C) and AQP4^−/−^ **D** tissues. Red arrows indicate the directionality of the central microtubular pair of each cilium in **C** and **D** (C’ and D’, respectively). In the upper right corner, a diagram is shown with the mean angles calculated for the analysed samples (N = 3). These data were used to construct histograms reflecting angle distributions in both WT **E** and AQP4^−/−^ **F** mice tissues. Scale bars = 5 µm (**A** and **B**) and 200 nm (**C** and **D**)

To study whether the ependymal cells of the AQP4^−/−^ mice also showed defects in achieving a correct orientation of the basal bodies and the central microtubular structure of the cilium, we performed transmission electron microscopy. The images were acquired from cilia identified at the ependymal edge (Figs. 5A and B), and the captures were taken at higher magnifications (Figs. 5C and D) to help recognise the direction and angle of vectors that join the pair of central microtubules (central apparatus). We verified that the mean deviation of the cilia was slightly higher in the ependymal cells of AQP4^−/−^ mice (Figs. 5C´ and D´), with mean deviations of 11.47° ± 0.54, 2° above the mean value for the WT (9.44° ± 0.27, p < 0.05). The angular deviation values for the quantified cilia are represented in the histograms shown in Figs. 5E and F. Note the greater dispersion of values for cells from the AQP4^−/−^ mice with respect to the greater homogeneity in the WT mice (higher frequencies for points close to 0° deviation).

### Comparison of the contractile function of ependyma in the cerebral aqueduct of WT and AQP4^−/−^ mice

**Fig. 6:**
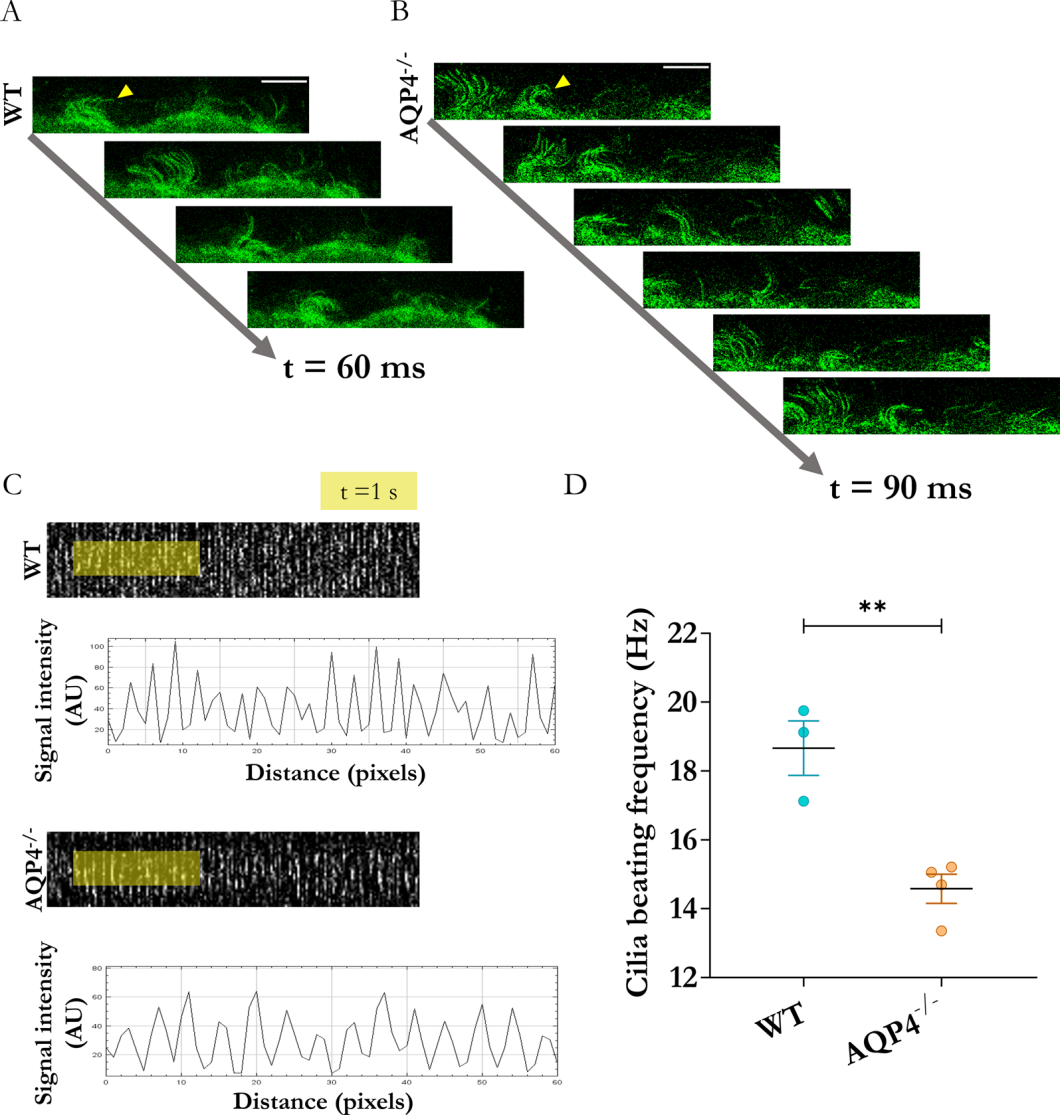
Study of ciliary beating frequency of ependymal cells in the aqueduct using ex vivo confocal microscopy. Frames from recorded videos of dissected tissue of interest from WT **A** and AQP4^−/−^ **B** mice at P11. In these examples, an increased time is required to complete one ciliary beat cycle in the transgenic mice compared with the WT (evaluated in representative ciliary tufts, yellow arrowheads). Scale bars = 10 µm. **C** Representative kymographs of ciliary beating rhythm in evaluated regions (below kymographs, intensity signal profiles obtained in the yellow area are shown, where each peak indicates each ciliary beat). AU = arbitrary units. **D** Quantification of mean frequency values and comparison between the two conditions. N = 3–4 per group. Data are presented as mean ± SEM. Unpaired Student’s t-test employed for statistical significance analysis. **p < 0.01

To search for ependymal functionality in our mice, we employed ex vivo microscopy strategies, which enabled the recording of mechanical activity in the ependymal cilia. Videos were acquired in various fields (see examples in Figs. 6A and B). Using kymographs, the ciliary beat frequencies were determined in the WT and AQP4^−/−^ mice (Fig. 6C). The quantifications showed a 20% decrease in the mean contraction rate in the ependymal cells of the AQP4^−/−^ mice compared with the WT mice (** p < 0.01).

### Validation of the transcriptomic analysis results of genes related to the immune function and presence of CD11c + microglial cells

As indicated previously in Fig. 2C, the Affymetrix analysis showed extensive enrichment in the gene profile of immune cell populations in the ependymal tissue of the AQP4^−/−^ mice. To corroborate this differential expression, validations were conducted using qPCR in independent biological samples. A selected group of genes that showed the highest differential expression after bioinformatic analysis were selected for RT-qPCR amplification. The results shown in Supplementary Fig. 1A confirm the significantly higher expression (** p < 0.01) of several of these markers in the tissues of the AQP4^−/−^ mice. Increments of more than 10 folds (12.80 ± 3.24 in AQP4^−/−^ and 1.26 ± 0.38 in WT) were observed in the genes encoding secreted phosphoprotein 1 (Spp1), also known as osteopontin, and transmembrane glycoprotein NMB (Gpnmb). A greater expression (fivefold, 5.34 ± 0.97 in AQP4^−/−^ compared to 1.14 ± 0.53 in WT, ** p < 0.01) was observed for the gene encoding the surface marker for dendritic cells Cd11c + (Itgax), and a somewhat lower (3.6 fold, 3.65 ± 0.81 in AQP4^−/−^ and 1.05 ± 0.15 in WT, ** p < 0.01) but still significantly greater expression of the macrophage marker (Cd68) was observed in the AQP4^−/−^ mice compared with the WT mice.

Given the predominant role that microglia play in CNS immunity, we further evaluated the expression of typical genes considered microglial marker genes such as those coding for the purinergic receptor P2Y12 (P2ry12), the transmembrane protein 119 (Tmem119) and the transcriptional regulator SALL1 (*Sall1*), as microglial signature genes. Interestingly, a lower expression was observed for the first two genes (Supplementary Fig. 1B) in the AQP4^−/−^ mice, suggesting that an immune cell type different from the homeostatic microglia widely described in the CNS was probably responsible for this expression pattern.

### CD11c + postnatal microglia in the brain aqueductal region

**Fig. 7:**
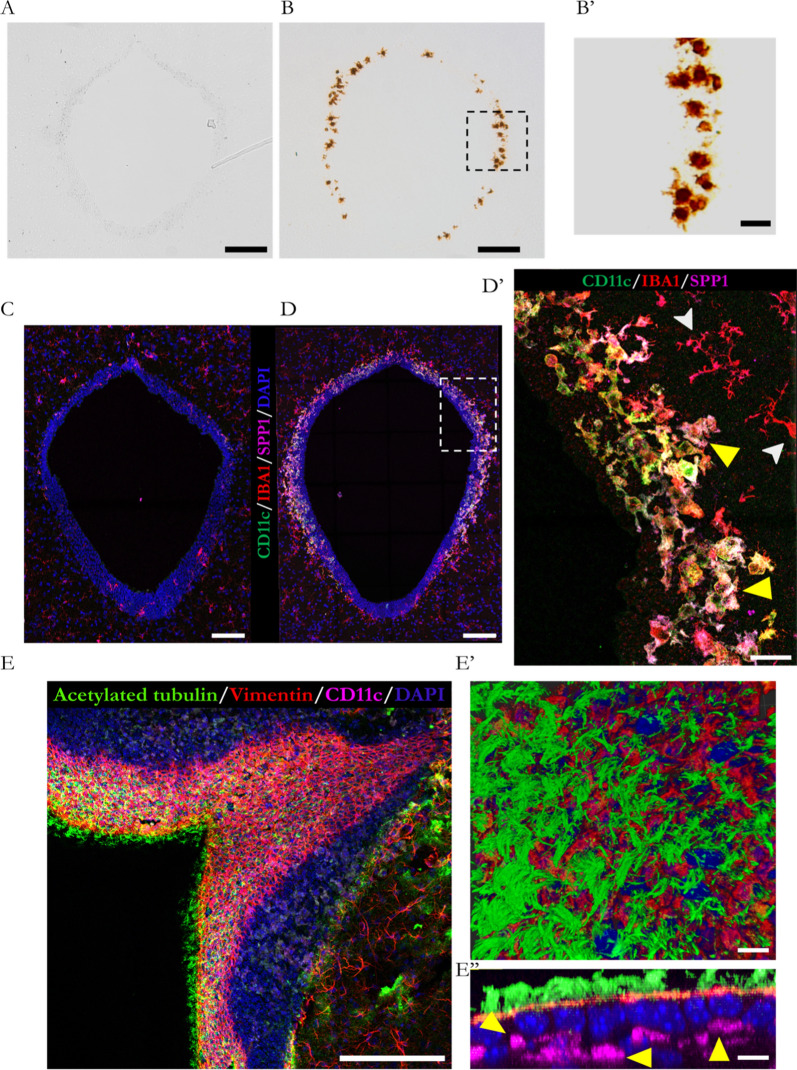
Histological characterization of the increased Osteopontin expression in the cerebral aqueduct of the AQP4^−/−^ mice at P11. Detection of Spp1 mRNA by ISH in coronal sections of the aqueduct from WT **A** and AQP4^−/−^ **B** mice. (B’) Magnification of the cerebral aqueduct edge with cells positive for chromogen. Immunostaining assays against gene-overexpressed markers SPP1 and CD11c, as well as characteristic microglial marker IBA1 in brain coronal sections of WT **C** and AQP4^−/−^ **D** mice at P11. **D**’ Magnified image of a cerebral aqueduct region with cells positive for all three markers (yellow arrowheads) and exclusively IBA1 + cells (white arrowheads). To determine the position of these microglial cells relative to the ependymal cells, fluorescence immunolabeling was performed in sagittal brain sections including the cerebral aqueduct region of the AQP4^−/−^ mice **E**. Generation of three-dimensional volumes using Imaris software depicting the appearance of ependymal epithelium observed on the superficial plane (E’) and perpendicular to it (E’’). Note in (E’’) the CD11c + microglial cells arranged in proximity to the basal face of the ependymal cell membranes. Scale bars = 150 µm **A**, **B**, **C**, **D** and **E**, 20 µm (B and D’) or 10 µm (E’ and E’’). N = 5 samples per condition

To identify the cells responsible for the expression (in the periaqueductal tissue) of immune function-related genes, we analysed the expression of *Spp1*, given that this gene showed the highest overexpression in the tissue. To this end, we performed in situ hybridisation. The results (Figs. 7A and B) revealed the presence of *Spp1* mRNA labelling exclusively in the tissue of the AQP4^−/−^ mice. A closer observation revealed the presence of the transcripts inside cells with spherical morphology and small branches arranged at the edges of the aqueduct (Fig. 7B'). This morphology was similar to that of the amoeboid microglia described during the early phases of postnatal development (P3 to P7) in white matter regions (corpus callosum and cerebellum) that also show *Spp1* as a representative gene marker [34, 35]. To further characterise these cells and according to the description by Wlodarczyk et al. [36], we performed immunofluorescence analysis against markers IBA1, CD11c and SPP1 (Fig. 7C, D and D´) to better characterise the subtype of microglial cell population observed in the aqueduct of the P11 AQP4^−/−^ mice. The results showed the co-localisation of the 3 markers in a cell type located at the limits of the aqueductal ependymal epithelium of the AQP4^−/−^ mice (Fig. 7D). In contrast, these cells were not identified in the tissue from the WT mice (Fig. 7C). Using higher magnification micrographs of the region of the AQP4^−/−^ mice (Fig. 7D'), it was possible to distinguish between IBA1 + /CD11c + /SPP1 + microglial cells with rounded morphology and few extensions (yellow arrows) closer to the ependymal edge, in contrast to IBA1 + /CD11c-/SPP1- (with narrower bodies and numerous branches) more integrated into the brain parenchyma (white arrows). To discern between a subependymal or supraependymal location for these cells, immunofluorescence assays were performed against the CD11c receptor and the characteristic ependymal markers vimentin and acetylated tubulin (specific for cilia) (Fig. 7E). Magnified images were acquired from the aqueductal ependymal tissue of the AQP4^−/−^ mice (Fig. 7E´), and three-dimensional reconstructions were performed that reproduced the labelling obtained (Fig. 7E´´). As can be seen in the micrograph that cuts perpendicular to the surface of the aqueduct (Fig. 7E´´), the microglial cells were always delimited within the brain parenchyma, in areas close to the basolateral membrane of the ependymal cells (yellow arrows). In these images, we also observed a significant closeness between the two cells (ependyma and microglial CD11c + cells), leading us to hypothesise a plausible functional interaction between the two cell types. Lastly, no CD11c + cells were identified on the ependymal surface, which was dominated by the ciliary structures. Therefore, these results identify microglia as the periaqueductal cells responsible for Spp1 production, ruling out other explanations that could imply, in certain cases, the arrival of cells such as macrophages to the aqueductal zone.

### 8-Time course of CD11c + microglia expression in the brain aqueduct region

**Fig. 8:**
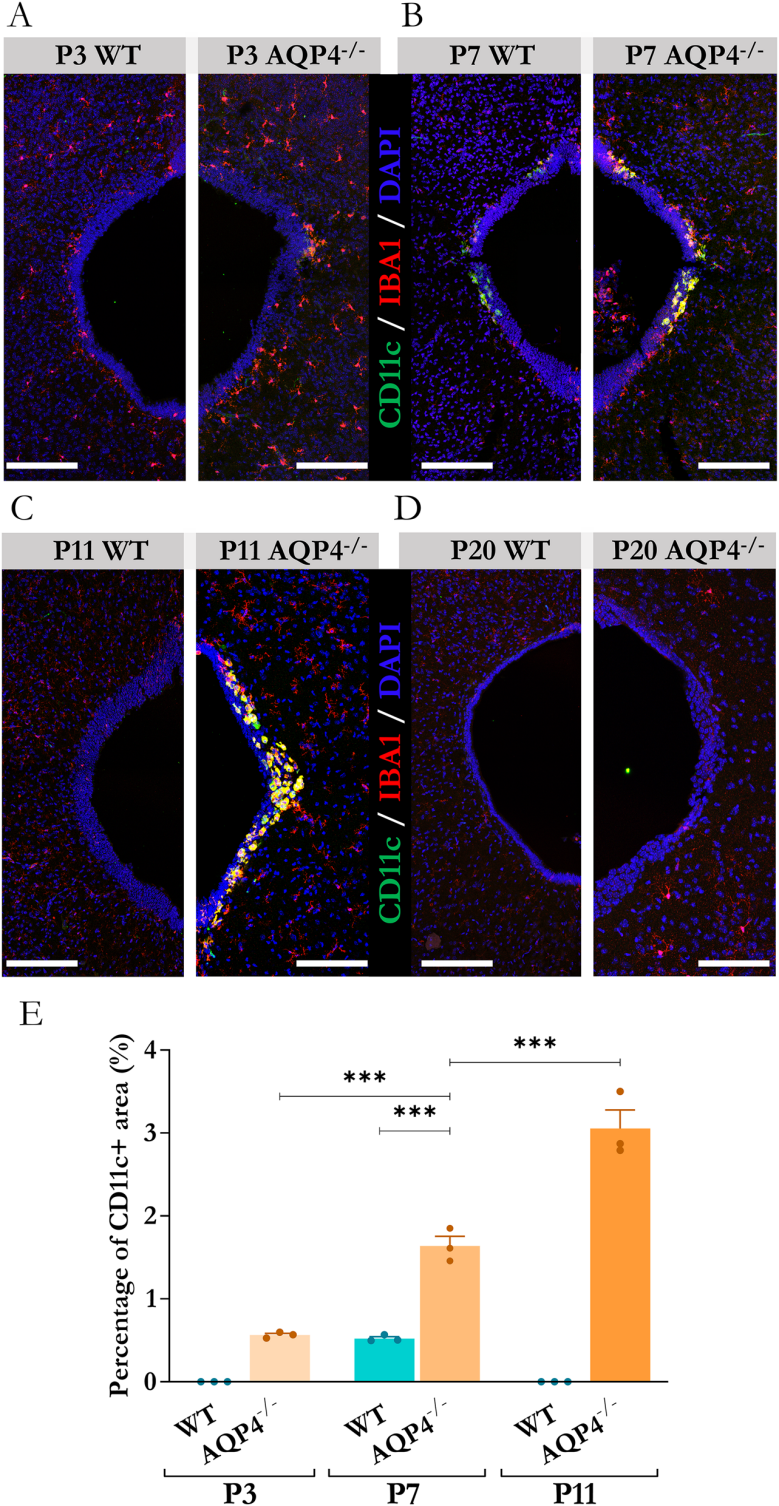
Evaluation of the temporal appearance of CD11c + microglial subtype in the cerebral aqueduct during early postnatal period. Compositions of confocal microscopy images to comparatively determine the presence of CD11c + microglial cells (WT vs AQP4^−/−^) at developmental stages P3 **A**, P7 **B**, P11 **C**, and P20 **D**. Scale bars = 150 µm. **E** Quantifications of microglial cell abundance in the evaluated sections. N = 3 samples per condition, from N = 3 animals at the different developmental stages. Data are presented as mean ± SEM. Significant differences between groups were assessed using one-way ANOVA followed by Tukey’s post hoc test (***p < 0.001)

To investigate whether the identified microglial presence was the result of the non-physiological absence of AQP4 or whether it was related to specific phases of perinatal neurodevelopment, we performed a time course for the expression of these CD11c + cells throughout the first month of life of the mice. Immunofluorescence assays against CD11c and IBA1 markers were performed in the periaqueductal tissues of the mice at specific stages of postnatal development (P3, P7, P11, and P20) (Fig. 8). The results demonstrated that, in the AQP4^−/−^ mice, a positive signal for CD11c expression was detected from stage P3 (Fig. 8A) to stage P11 (Fig. 8C), to ultimately drop to negligible levels by P20 (Fig. 8D). CD11c + cell expression was also detected in the aqueduct of the WT mice, but the time course for the onset of this cell type in these mice was more limited, observed only at P7 (Figs. 8A and B), and the abundance of CD11c + cells was lower than that detected in the AQP4^−/−^ mice. Quantification of the labelling signal (Fig. 8E) corroborated the greater abundance of the CD11c + population in the transgenic mice at any point with respect to the WT mice. A gradual increase between stages P3, P7, and P11 was observed (p < 0.001), with P11 being the point of development at which the maximum labelling signal was detected.

### 9- Comparative analysis of aqueduct CD11c + microglia and other microglia populations

To better understand whether the presence of CD11c + microglia is associated with the normal neurodevelopment process or is part of a reaction response to the abnormal absence of AQP4 during development, the gene profiles of microglial subtypes in other studies were compared and related according to the expression levels of the most representative genes to the gene profile of CD11c + microglia found in the aqueduct (AQP4-Aq) in the mice of the present study.

The correlation matrix (Supplementary Fig. 2A) shows that the greatest genetic similarity of the microglial subtype identified in our mice (AQP4-KO-Aq) was with that described for newborn populations in a neurodevelopmental situation (groups CD11c and PAM, correlation levels of 0.73 and 0.69, respectively) and not with those associated with pathological processes. The degree of homology exhibited with populations related to pathological situations (disease associate microglia (DAM), amyloid precursor protein (APP), neurodegenerative microglia (MGnD) or lipid-droplet accumulating microglia (LDAM) subtypes) or ageing (Aged) was lower. To provide additional evidence of the association between these microglia and neurodevelopment (rather than with disease), we analysed the cellular ultrastructure of these microglia using TEM after immunolabeling against IBA1. Using TEM, we detected the accumulation of gold particles in cell subtypes that, due to their location (close to the basal membrane of the ependymal cells) and morphology (Supplementary Fig. 2B), could correspond to the identified microglial type. Images taken at high magnification revealed striking enrichment in the cellular inclusion bodies (phagocytic vesicles), as well as mitochondrial content (Supplementary Fig. 2B'), two of the fundamental characteristics that have been described for CD11c and PAM microglia, with both microglia linked to neurodevelopment [34, 36].

## Discussion

### Structural and functional development of aqueductal ependymal cells in the absence of AQP4 expression

Located at the narrowest part of the CSF circuit, the Sylvian aqueduct is the most common site of intraventricular blockage of the CSF, and its stenosis is responsible for a large number of cases of hydrocephalus in children and adults [54]. As in the rest of the ventricular compartments of the brain, the ependymal cells that cover the surface of the aqueduct have a multiciliated nature with a profile of receptors, junctional complexes, and cellular channels that are fundamental for its functions in the brain. After differentiation from radial glia in the perinatal stage, ependymal cells activate a specific transcriptional programme between the first and third postnatal weeks, which concludes with the acquisition of the cellular characteristics that give them their mature functional capacity [23]. During this ependymal maturation interval, we confirmed an increase in AQP4 expression (Fig. 1), which suggests that this protein plays an important role in various neurodevelopment processes in mice, as proposed in our previous study [45].

The present study, which employed WT mice as controls and AQP4^−/−^ mice (AQP4-KO) lacking full AQP4 expression, analysed the role of the AQP4 protein in the formation and development of aqueductal ependymal cells. The Affymetrix analysis revealed that the gene expression associated with ependymal tissue, ciliar formation, and ependymal development was reduced in the AQP4^−/−^ mice (Fig. 2A-2C). The qPCR validations corroborated these changes, revealing defects in the expression of genes involved at specific points of ependymal development (Fig. 3), such as the downregulation of transcription factors (FoxJ1, Rfx4) and lower expression of genes necessary for constituting the central microtubule pair (Cfap43, Cfap69 or Spag16) in the cilia, as well as a reduction in the expression of genes coding for binding proteins of the cell junctions that line the ependyma (Cdhr4).

Through ciliary beating, multiciliated ependymal cells contribute significantly to the movement of CSF. One of the key components of this function is the rotational and translational PCP of these ciliated cells. Defects in the PCP of ependymal cells can result in abnormal propulsion or accumulation of CSF and consequently hydrocephalus [23]. We therefore examined whether the altered gene profile observed in the Affymetrix analysis modified the PCP of the multiciliated ependymal epithelium in the AQP4^−/−^ mice. To this end, we compared, in the AQP4^−/−^ and WT mice, the position of the basal bodies on the apical surface of the cell (translational PCP) and the angle of individual basal bodies with respect to their long axis (rotational PCP), as previously shown by Mirzadeh et al. [54]. Our results (Fig. 4) led to the conclusion that the degree of grouping and the orientation of the basal body path with respect to the centre of the apical surface of the cell was much more homogeneous in the WT mice than in the AQP4^−/−^ mice. The angle of the vector that connects the centres of the entire cell area with that of the area where the ciliary basal bodies were located was significantly more similar between neighbouring cells in the epithelium of the aqueductal ependyma of the WT mice than in the AQP4^−/−^ mice, demonstrating significant disorganization in the position of the basal body path in the KO mice. A significant reduction in the surface area occupied by the apical membrane of the ependymal cells was also observed in the AQP4^−/−^ mice. This reduced surface for anchoring of the ciliary basal bodies could be partly responsible for the poorer translational PCP observed in the transgenic mice. During the development of the ependymal cell, it is essential that the apical cellular surface increases in measure to adapt to the increase in ventricular size [55]. A smaller ventricular size has been reported in AQP4^−/−^ mice, [5, 14], consistent with the reduced PCP seen in our study, for ependymal cells developed in the absence of AQP4 expression (AQP4^−/−^). Additionally, SEM observations of the ependymal epithelium (Fig. 4D-E) showed greater dispersion of the ciliary tufts and poorer grouping of the cilia in the KO mice, consistent with a PCP alteration of the ependymal tissue. The loss of the symmetrical arrangement of the ciliary protrusions that characterise this epithelium has frequently been associated with defects in the establishment of the convective CSF currents that cells generate in the ventricular cavities and therefore with hydrocephalus [56]. Similarly, the establishment of this flow and the maturation of the cilia are elements that enhance each other (the greater convective flow promotes the grouping of cilia and vice versa), as various studies have indicated [57, 58].

The effectiveness of a proper CSF flux also depends on the capacity of ependymal tissue to achieve a uniform orientation of the cilia, that is, to develop a correct rotational PCP. Using TEM, our study showed that the orientation angles of the central microtubule pairs showed greater dispersion in the tissue of the AQP4^−/−^ mice (Fig. 5), which shows, in concordance with the previously discussed alterations in the translational PCP, that a developmental defect occurs in the cilia of the aqueductal ependymal cells in the AQP4^−/−^ mice. As indicated previously [59], rotational PCP requires that internal (communication between the basal bodies of each cell) and external mechanisms (intercellular communication between neighbouring cells) are activated in ependymal cells to achieve a CSF orientated synchronous and homogeneous shake. Therefore, a structural deficit in the development of the ciliar ependyma, which would lead to a disturbance in the ciliary beat and thus to the rhythmic movement of the CSF in the AQP4^−/−^ mice, would also be responsible for disorders such as the hydrocephalus reported in a number of these mice [14].

Furthermore, a detailed TEM evaluation of the intercellular unions between the ependymal cells of each genotype (WT and AQP4^−/−^) reveals enrichment of gap and adherents junctions in the WT mice, in contrast to the predominance of occludent and shorter gaps unions observed in the AQP4^−/−^ mice (Supplementary Fig. 3), all of which suggest a less mature ependyma epithelium in the KO mice [60]. Immunohistochemistry analysis [14] has demonstrated that deletion of AQP4 results in decreased expression of the gap junction protein connexin 43 in ependymal cells of KO mice, which is consistent with our findings. The presence of gap junctions has been associated with functions in the ciliary constitution, through changes such as cytoskeleton remodelling and the presence of contact points with the microtubules on the cytosolic side [61]. Such differences in intercellular binding unions can also determine functional changes in the nature of the ependymal-CSF barrier of the AQP4^−/−^ mice. A singular feedback mechanism seems to be in play here, such that a change in AQP4 expression produces changes in the expression of cytoskeleton components of the cell membrane (as shown in this study) and vice versa; alteration of cytoskeleton elements and scaffold proteins would be decisive for the location of the channel at specific points of the cell membrane [62, 63].

To demonstrate whether the previously described structural alterations, namely, changes in the apical PCP of the ependyma, differences in the size of the apical surface of the cells, and changes in the prevalent type of intercellular unions affect tissue motility, we evaluated the contractile activity of these ciliary cells by monitoring the beating frequencies of ciliary cells in ex vivo experiments. Our results (Fig. 6) demonstrated poorer contractility capacity after determining that the ciliary beat frequencies in the AQP4^−/−^ mice were clearly reduced compared to those of the WT mice. In the AQP4^−/−^ mice, reduced ventricular size, reduced intraventricular pressure [5], and a significant increase in extracellular space [64] were observed. The results of the study by Gomolka et al.[65] support these changes and show that, in the absence of AQP4, greater stagnation of interstitial fluid due to blockage of glymphatic transport would lead to a reduction in CSF circulation and alter its homeostasis.

Our study determined that the reduction in the expression levels of genes involved in ciliary development was incomplete in the AQP4^−/−^/C57BL/6 mice, which could help to explain why we did not observe a hydrocephalus phenotype in our animal, as has been observed by other authors in the AQP4^−/−^-CD1 mice [14, 30]. Furthermore, the multiplicity of transcription factors, e.g., those of the Rfx family (Rfx1-4), as well as the presence of numerous mechanisms and regulatory interactions between the products of genes involved in cilia development [66], which could lead to compensation for the defects of genes identified in our study, could also help prevent the complete aqueductal stenosis, preventing the development of an obstructive hydrocephalus in our AQP4^−/−^ mice.

### Presence of a CD11c + microglia in the development of aqueductal ependyma

The transcriptomic study of the periaqueductal tissue also highlighted increases in the expression levels of markers linked to an immune activation profile in the P11 AQP4^−/−^ mice (Fig. 2C). Genes such as Spp1, Cd68, and Gpnmb were overexpressed in the KO mice, which prompted the search for an immune cell type responsible for their expression in the aqueductal tissue of these mice. The validation of the changes in the expression levels of a number of these genes (Supplementary Figs. 1A and 1B), as well as the clear periaqueductal location of the Spp1 mRNA and the Osteopontin protein (Fig. 7) showed that a CD11c + microglial population was responsible for the identified gene profile in the AQP4^−/−^ mice. This CD11c + microglia with amoeboid morphology (Fig. 7D') in intimate contact with the basolateral membrane of ependymal cells (Fig. 7E-E´-E´´) was exclusively observed in the aqueduct of AQP4^−/−^ mice at P11 and was completely absent in the WT mice of the same age. The presence of microglial cells in the subependymal layers of the ventricular system of neonatal rabbits and rats has previously been reported and associated [67, 68] with phagocytic activity and high proliferative capacity. These microglia, then called”hyperpendimal”, could be equivalent to the microglia in the present study in the periaqueductal zone of the AQP4^−/−^ mice. Of the various postnatal development points (P3–P20) (Fig. 8), subependymal periaqueductal microglial cells CD11c + were detected in the WT mice only at P7. Their presence lasted longer and had a greater magnitude along the postnatal period analysed in the AQP4^−/−^ mice, probably indicating an abnormal physiological state of the tissue in this zone and/or a relevant function of this particular microglia cell type in acquiring the normal development of the ependymal epithelium. In addition to development, various studies have reported that certain insults, such as the intraventricular injection of toxic compounds, can cause massive infiltration of these microglial cells into subependymal tissue as an acute response [69]. Having demonstrated structural and functional alterations in the development of the aqueductal ependyma epithelium of our study AQP4^−/−^ mice, we explored whether CD11c + microglial expression at P11 could arise as a response to damage or as an expansion of the neurodevelopment phase during which the final maturation of the ependymal epithelium occurs. The comparative study of the transcriptomic fingerprints of the cell subtype found in the aqueduct of the AQP4^−/−^ mice, called AQP4-Aq (Supplementary Fig. 2), against those of microglial populations described by other authors [34–36, 38–41, 70] leads us to conclude that the nature and presence of these CD11c + microglia are related to the mice’s neurodevelopment process, more than to a defence response against harm. Characterisation of the various microglia subtypes grouped under the postnatal profile category of CD11c, PAM, and ATM [34–36] coincides primarily with the gene expression repertoire of our CD11c + microglia AQP4-Aq. An important phagocytic capacity of these cells during the development of tissues around which these microglia are strategically placed has also been reported [71]. This property agrees with the ultrastructural characteristics observed in our study (Supplementary Figs. 2B and B´) in which a high content of potentially phagocytic vesicles was observed in microglial AQP4-Aq cells, which would fit the previously described gene profile.

Another characteristic of AQP4-Aq microglial cells is the high expression of factors associated with development, such as IGF1 and Osteopontin, both of which have demonstrated the ability to exert neurotrophic activity in neural populations [72] and both having receptors expressed by ependymal cells [24]. Osteopontin, a pleiotropic molecule susceptible to multiple modifications, could act on the β1 integrin receptors on the basal surface of ependymal cells, triggering the activation of various signalling cascades. Interestingly, several studies have also proposed possible molecular interaction routes between osteopontin and AQP4 [73], which together with our results leads to the conclusion that osteopontin overexpression could arise as a compensatory mechanism for the loss of AQP4 in the ependymal cell. Osteopontin might be contributing to the rescue of aqueductal ependyma in AQP4^−/−^ mice, preventing its occlusion and the development of obstructive hydrocephalus. The absence of this compensatory mechanism, for unknown reasons, could occur in a small number of AQP4^−/−^ mice in which congenital hydrocephalus has been reported [14, 30].

## Conclusions

Our results show for the first time that the lack of AQP4 expression in the aqueductal ependyma changes the expression of numerous other genes, which in turn alter the PCP of the ciliated aqueductal ependymal cells and reduce ciliary beating velocity, affecting the normal circulation of CSF through the aqueduct. Changes in the normal circulation of CSF will lead to the onset of hydrocephalus under certain conditions in a few mice. We also identify for the first time a CD11c + microglial cell population underlying the periaqueductal ependyma that appears during the postnatal (P7) period of healthy WT mice. In mice lacking AQP4, a prolonged and stronger presence of these CD11c microglia (AQP4-Aq) will be essential for compensating for the normal development of the ependymal epithelium, thus preventing the onset of hydrocephalus.

### Supplementary Information

Supplementary material 1. Figure 1. Analysis of the differential expression of genes associated with immune function and microglial cells. (A) Gene expression analysis by RT-qPCR assessing the expression levels for immune system-related genes identified in the microarray transcriptome study. (B) Gene expression levels of microglial markers quantified by RT-qPCR and compared between WT and AQP4^-/-^ tissues. N=6–8 per condition. Data are presented as mean ± SEM. Unpaired Student’s t-test employed for statistical significance analysis. **p<0.01

Supplementary material 2. Figure 2 Comparative study of the microglial gene profile identified in the AQP4^-/-^ cerebral aqueduct and characterization of its ultrastructure. (A) Correlation matrix of the gene profile constructed from data obtained by microarray and those provided by studies defining various microglial types through transcriptomic approaches. (B) Electron micrographs of aqueduct sections subjected to immunogold immunolabeling assay against IBA1. In the magnified image (B’), the presence of gold colloid particles (yellow arrowheads) is marked, as well as cellular structures (inclusion bodies or I.B. and mitochondria or M.). Scale bars = 1 µm (B) or 350 nm (B’)

Supplementary material 3. Figure 3. TEM evaluation of cellular junction complexes in the ependyma of the cerebral aqueduct. Representative transmission electron micrographs showing various junction structures between ependymal cells of WT (A) and AQP4^-/-^ (B) mice. Higher magnification images reveal different types of union complexes (AJ=adherens junctions; TJ=tight junctions and GAP) in both conditions (A’ and B’). N=3 per group. Scale bars = 1 µm (A and B) and 500 nm (A’ and B’)

## Data Availability

https://idus.us.es/browse?type=author&value=Echevarr%C3%ADa+Irusta%2C+Miriam

## Abbreviations

AQPs: Aquaporins
AQP1/4: Aquaporin-1/and -4
ATM: Axon tract-associated microglia
BBB: Blood brain barrier
BDNF: Brain-derived neurotrophic factor
CC: Corpus callosum
CD11c: Cluster of differentiation 11c, Microglial marker- αX integrins receptor
CNS: Central nervous system
CSF: Cerebrospinal fluid
DAM1/2: Disease-associated microglia 1/2
DAPC: Dystrophin-associated protein complex
DAPI: 4',6-Diamidino-2- phenylindole, DNA marker
FC: Fold change
GFAP: Glial fibrillary acidic protein
GO: Gene ontology
GOBP: Gene ontology for biological processes
GOCC: Gene ontology for cellular components
GS: Gene set
IBA1: Ionised calcium-binding adapter molecule 1, microglia marker
IGF1: Insulin-like growth factor 1
MBP: Myelin basic protein
MGnD: Neurodegenerative microglia
PAM: Proliferative region-associated microglia
PCP: Planar cell polarity
RT-qPCR: Real-time quantitative polymerase chain reaction
SEM: Scanning electron microscopy
SPP1: Osteopontin, secreted phosphoprotein 1
TEM: Transmission electron microscopy
WT: Wild mice, Wild type
