# Supplementary figures and images for: Impact of aquaporin-4 and CD11c + microglia in the development of ependymal cells in the aqueduct: inferences to hydrocephalus
Source: Fluids Barriers CNS. 2024 Jul 2;21:53. doi: 10.1186/s12987-024-00548-2 (PMC11221146; doi:10.1186/s12987-024-00548-2)

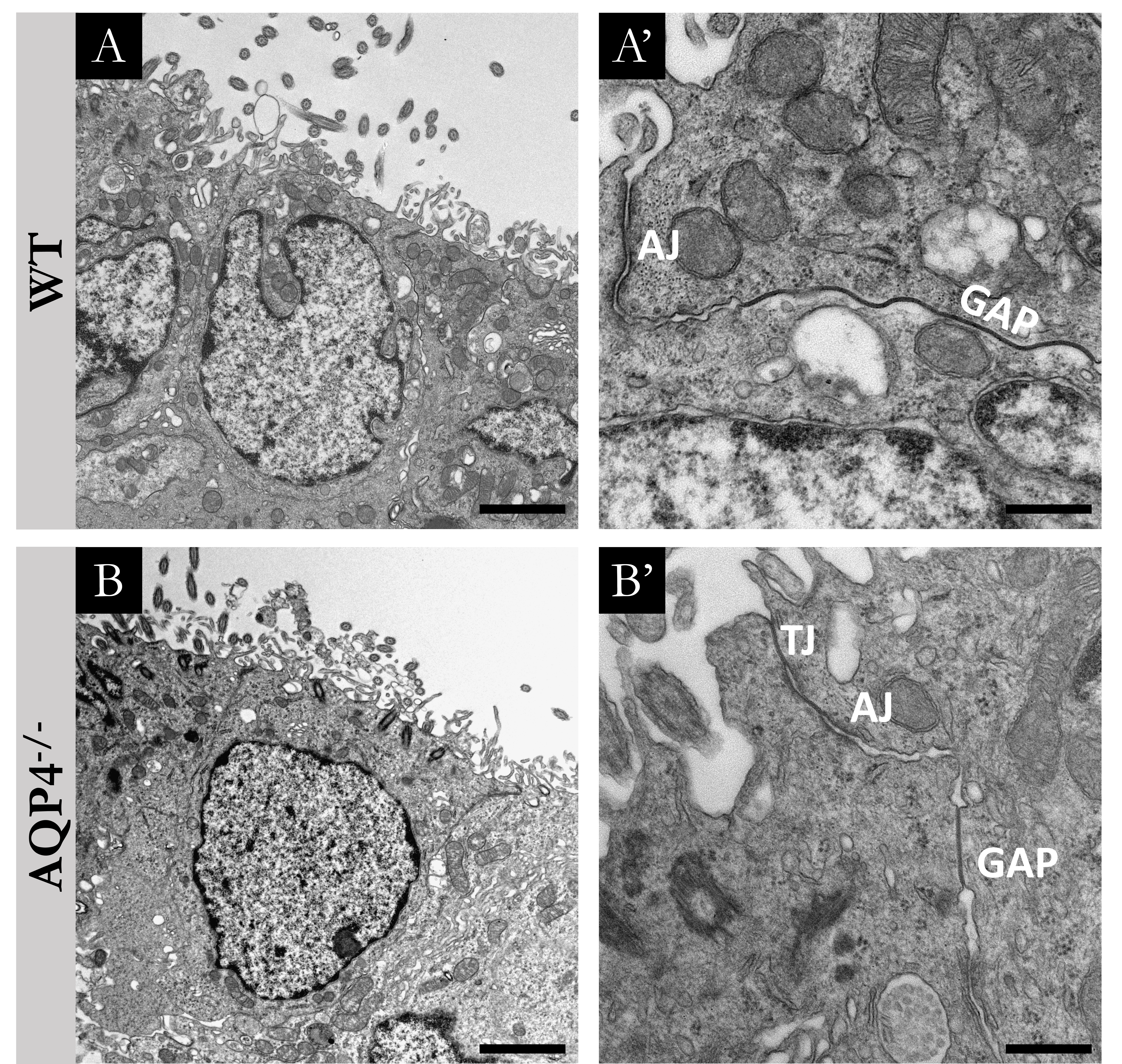

Supplement: Supplementary file 3 — Supplementary material 3. Figure 3. TEM evaluation of cellular junction complexes in the ependyma of the cerebral aqueduct. Representative transmission electron micrographs showing various junction structures between ependymal cells of WT (A) and AQP4-/- (B) mice. Higher magnification images reveal different types of union complexes (AJ=adherens junctions; TJ=tight junctions and GAP) in both conditions (A’ and B’). N=3 per group. Scale bars = 1 µm (A and B) and 500 nm (A’ and B’) [file 12987_2024_548_MOESM3_ESM.tif]
